# Unprovoked seizures in multiple sclerosis: Why are they rare?

**DOI:** 10.1002/brb3.726

**Published:** 2017-05-24

**Authors:** Anamarija Kavčič, Werner E. Hofmann

**Affiliations:** ^1^ Gemeinschaftspraxis Dr. Hofmann & Olschewski Aschaffenburg Germany

**Keywords:** blood–brain barrier, multiple sclerosis, neurodegeneration, neuroinflammation, seizure

## Abstract

**Introduction:**

The frequency of seizures in patients with multiple sclerosis (MS) ranges from 1.5% to 7.8% and is considerably more common than chance events. The etiopathogenesis of seizures in MS is still poorly understood.

**Method:**

A review of the literature on seizures and MS using PubMed.

**Results:**

Cortical gray matter involvement appears to be an all‐too‐common pathological finding in MS to play a primary role in the pathogenesis of seizures in MS patients. There is no clear relationship between seizures and the severity of MS. In approximately 10% of cases, a seizure is actually an initial neurological symptom of MS.

**Conclusion:**

Searching for coherence in the occurrence of unprovoked seizures in MS directs attention to the dichotomy in MS pathology characterized by a complex intertwining of neuroinflammatory and neurodegenerative processes. The appearance (or nonappearance) of seizures in MS in relation to disease activity and disease progression indicates a distinct clinical phenotype of MS that opens up new perspectives in MS research.

## 1 INTRODUCTION

Seizures were recognized as a clinical manifestation of multiple sclerosis (MS) by the German pathologist, neurologist and internist Wilhelm von Leube as early as 1871 (Leube, 1871). Subsequent studies have shown that seizures occur during the course of MS more commonly than chance events (Kelley & Rodriguez, 2009; Marrie et al., 2015). According to recent epidemiological studies, the frequency of seizures in MS patients varies between 1.5% and 7.8% (Lund, Nakken, Edland, & Celius, 2014).

The etiopathogenesis of seizures in MS is still poorly understood (Ciccarelli et al., 2014). Considering the frequency of seizures in MS, which is relatively low, the involvement of cortical gray matter seems to be too common a pathological finding in MS to play a central role in epileptogenesis in MS patients (Barkhof, 2002; Haider et al., 2016; Van Munster, Jonkman, Weinstein, Uitdehaag, & Geurts, 2015).

## 2 METHOD

We reviewed the literature on seizures and MS using PubMed.

## 3 RESULTS

### 3.1 Fulminant MS versus nondisabling MS

In fulminant MS, the most malignant form of MS, seizures do not always occur (Blunt, Boulton, Wise, Kennard, & Lewis, 1994; Johnson, Lavin, & Whetsell, 1990; Nozaki & Abou‐Fayssal, 2010; Suzuki et al., 2013; Tutuncu et al., 2011). Even in fatal cases of fulminant MS, seizures are not always observed (Blunt et al., 1994; Johnson et al., 1990; Suzuki et al., 2013). A seizure does not usually signal fulminant MS (Elenein et al., 2011; Gupta, Vasishta, Kharbanda, Vyas, & Prabhakar, 2011; Rohani & Ghourchian, 2011), and seizures do not ordinarily appear in the early days of fulminant MS (Elenein et al., 2011; Rohani & Ghourchian, 2011).

On the other hand, cases of nondisabling MS with seizures are not infrequent (Kelley & Rodriguez, 2009; Striano et al., 2003). In actuality, a seizure is sometimes—in approximately 10% of MS cases according to miscellaneous studies—an initial neurological symptom of MS (Catenoix et al., 2011).

## 4 DISCUSSION

The absence of a correlation between unprovoked seizures and the severity of MS may seem contradictory. However, increased seizure susceptibility in MS might be moderated, at least partly, by corticosteroids. These have been widely used in the treatment of acute exacerbations of MS since the early 1950s (Schmidt & Hoffmann, 2012). On the other hand, they have proved beneficial in some epileptic syndromes (Aarli, 2000). It is, therefore, possible that corticosteroids, owing to their anti‐epileptic properties, reduce seizure frequency in MS or even prevent seizures in some MS patients who are receiving high‐dose corticosteroid treatment.

Given that the precorticosteroid era was also the pre‐MRI era, it is not possible to elucidate the supposed anticonvulsant effect of corticosteroids in MS retrospectively. Diagnosing MS without MRI has always been a great challenge as well as a source of inaccuracy. Because of the undoubted beneficial effect of corticosteroids in the acute exacerbation of MS, it would be ethically unacceptable to study the subject prospectively, using a randomized controlled trial. Accordingly, corticosteroid therapy seems set to remain both a highly speculative and a highly effective treatment option for incurable MS.

### 4.1 Blood–brain barrier disruption: a crossing point between epilepsy and MS?

In recent years, many studies have shown that brain inflammation plays an important role in neuronal excitability and epileptogenesis (Aarli, 2000; Amhaoul, Staelens, & Dedeurwaerdere, 2014; Marchi et al., 2010; Ransohoff, 2009). Epileptiform activity seems to be facilitated by a breakdown of the blood–brain barrier (Ransohoff, 2009). This finding corresponds well with clinical observations of the significantly increased frequency of seizures in MS patients in comparison with the general population (Kelley & Rodriguez, 2009). However, given that the blood–brain barrier in MS is generally disrupted (Ciccarelli et al., 2014), it is quite surprising that less than 10% of MS patients experience seizures.

If we consider this paradox as a type of functional adaptation of the central nervous system in MS patients, we must ask what it is that protects so many MS patients from seizures.

Even when there were no efficient therapeutic options for MS, seizures were not observed in MS patients as a rule (Landtblom, Fazio, Fredrikson, & Granieri, 2010; Leube, 1871; Murray, 2009). Thus, one would assume that MS pathology per se could activate an adaptive mechanism that keeps the neuronal network stable in spite of a disruption in the blood–brain barrier.

Seizures usually emerge within the first decade of MS disease, i.e., 6.8 ± 6.1 years after the clinical onset of MS according to various population‐based cohort studies (Kelley & Rodriguez, 2009). This pattern of seizure occurrence in MS patients indicates that the human brain affected by MS can infinitely counteract increased seizure susceptibility. However, there are also other factors, including pulse steroid therapy and health‐oriented lifestyle, which can assist a human brain with a compromised blood–brain barrier in maintaining the stability of the neuronal network.

Nevertheless, the neural network in MS remains fragile. Unprovoked seizures may actually occur at any time in the course of MS regardless of the severity and activity of the disease (Kelley & Rodriguez, 2009).

Fragility of the neural network in MS patients without seizures (and in MS patients with seizures who are seizure‐free on medication) reflects the natural history of convulsive status epilepticus in MS patients as well. It is not uncommon for MS patients to experience a convulsive status epilepticus (Kelley & Rodriguez, 2009), and if it occurs, it can be difficult to treat (Kelley & Rodriguez, 2009).

### 4.2 Does neurodegeneration in MS finally diminish neural network excitability?

Considering the complexity of MS reflected in the paradoxical coexistence of degenerative and regenerative processes (Ciccarelli et al., 2014; Mahad, Trapp, & Lassmann, 2015), the relative infrequency of seizures in MS patients could also be viewed as a pseudoadaptation.

A hypothetical example of pseudoadaptation in MS regarding seizures would be decreased neural network excitability due to neurodegenerative processes, resulting in a widespread loss of neurons. This could be expected particularly in advanced MS.

The relative infrequency of epileptic seizures in MS patients with a great brain MRI lesion load (Barkhof, 2002) supports the above hypothesis.

The fact that seizures emerge sporadically in severe forms of MS does not exclude the hypothesis concerning decreased neural network excitability in advanced MS. Firstly, a late‐onset seizure in MS usually responds well to drug therapy (Kelley & Rodriguez, 2009); secondly, it is hardly certain if such seizures are actually unprovoked, i.e., related to MS pathology.

### 4.3 Cognitive dysfunction in MS is frequent, yet seizures are infrequent

Consideration of why seizures are a rather rare event in MS brings attention to the cognitive dysfunction in MS.

It is estimated that cognitive dysfunction occurs in up to 70% of MS patients (Rocca et al., 2015). The pathogenesis of cognitive dysfunction in MS remains controversial (Camp et al., 1999; Sumowski et al., 2014). There are no clear links between subcortical white matter pathology and cognitive dysfunction in MS (Kidd et al., 1999; Staff, Lucchinetti, & Keegan, 2009). However, cortical structural abnormalities of MS seem to be a satisfactory explanation for only some specific cognitive deficits observed in MS patients (Zarei, Chandran, Compston, & Hodges, 2003).

As a result of recent advances in brain‐imaging techniques, the visualization of MS lesions in vivo, especially cortical lesions, has improved dramatically (Liu et al., 2015; Rocca et al., 2015). Consequently, it has become evident that cortical changes in MS are neither an exception nor a characteristic of the advanced stages of MS (Haider et al., 2016; Rocca et al., 2015). In light of this, a high frequency of cognitive dysfunction in MS arises as a logical outcome. At the same time, it seems surprising that cortical lesions in MS do not usually trigger a seizure. Furthermore, it is astonishing to see how many MS patients with plenty of cortical lesions never experience a seizure.

### 4.4 Neuroinflammatory and neurodegenerative facets of MS

Considering the multifaceted nature of MS, the natural history of seizures related to MS cannot be anything other than multifarious.

In resolving apparent incompatibilities in the epileptogenicity of MS pathology, a dual approach, i.e., combining neuroinflammatory and neurodegenerative factors, corresponding to a dual pathology of MS (Ciccarelli et al., 2014), appears to be constructive. It can cover both scenarios: high epileptogenicity in MS pathology, most notably observed in MS patients for whom an epileptic seizure was a first symptom of MS; and low epileptogenicity of MS pathology, observed in MS patients with a high brain MRI lesion load who have never experienced a seizure.

Regardless of the origin of the inflammatory and neurodegenerative processes in MS, unprovoked seizures in MS could represent a dynamic interweaving of brain inflammation and neurodegeneration taking place in MS.

If we compare MS with Rasmussen′s encephalitis, an instructive example of chronic inflammation of usually one cerebral hemisphere, resulting frequently in pharmaco‐resistant focal seizures (Olson et al., 2013), it is evident that brain pathology of MS is in general considerably less proconvulsive than brain pathology in Rasmussen′s encephalitis. In particular, MS pathology can also exhibit high epileptogenicity, as observed in MS with unprovoked seizures as a first and foremost symptom of the disease.

Although MS and Rasmussen′s encephalitis are two different disease entities, whose etiology and pathophysiology are not fully understood (Ciccarelli et al., 2014; Olson et al., 2013), they share two conspicuous characteristics of brain inflammation, i.e., a chronicity and immunological background (Waisman, Liblau, & Becher, 2015). Given that the human brain has a limited repertoire for responses to injury and disease, a comparison of MS and Rasmussen′s encephalitis makes sense.

Thus, it can be said that MS with unprovoked seizures can be an indication of a dominance of brain inflammatory processes over neurodegenerative processes.

A diametrically opposite relationship between brain inflammation and neurodegeneration in MS, i.e., a predominance of neurodegenerative processes over the inflammatory processes, is to be expected, especially in MS with a high brain lesion load.

Other neurodegenerative diseases with prominent cortical pathology reveal a similar relative infrequency of seizures.

For example, in Alzheimer′s disease, which is a neurodegenerative disease with cortical pathology, especially in the temporal and limbic lobe, unprovoked seizures are uncommon, although they occur more frequently than in the general population (Born, 2015; Scarmeas et al., 2009). In corticobasal degeneration, another example of neurodegenerative disease with an extensive cortical pathology, unprovoked seizures are observed only rarely (Douglas, De Armond, Aminoff, Miller, & Rabinovici, 2009).

A striking discrepancy between cortical pathology and unprovoked seizures in neurodegenerative diseases supports the assumption that neurodegeneration per se is ultimately antiepileptogenic. It is possible that some stages of neurodegenerative processes act in a proconvulsive way. The occurrence of de novo seizures in patients with a neurodegenerative disease indicates precisely this phenomenon (Born, 2015; Scarmeas et al., 2009). However, when the progressive loss of structure or function of neurons due to neurodegenerative processes reaches its critical point, it is probably the neuronal network disintegration that prevents excessive and/or hypersynchronous activity of neurons in the brain.

## 5 CONCLUSION

The natural history of unprovoked seizures in MS seems illogical in the first instance. Considering the neuroinflammation and neurodegeneration observed in MS, high epileptogenicity of MS pathology could reflect a dominance of brain inflammatory processes over neurodegenerative processes. On the other hand, low epileptogenicity of MS pathology could indicate a predominance of neurodegenerative processes over inflammatory processes. Consequently, unprovoked seizures in MS open up new perspectives in MS research.

## CONFLICT OF INTEREST

The authors have declared that no conflict of interest exists.
